# Autonomic cardiac profile in male and female healthcare professionals with and without preschoolers: differences evidenced by heart rate variability analysis

**DOI:** 10.1038/s41598-022-18744-1

**Published:** 2022-08-25

**Authors:** Beatrice De Maria, Giuseppina Cassetti, Letizia Clementi, Valeria De Grazia, Monica Parati, Francesca Perego, Alberto Porta, Laura Adelaide Dalla Vecchia

**Affiliations:** 1 Istituti Clinici Scientifici Maugeri IRCCS, Milan, Italy; 2 Department of Electronics Information and Bioengineering, Politecnico di Milano, Milan, Italy; 3 MOX, Department of Mathematics, Politecnico di Milano, Milan, Italy; 4 CHDS, Center for Health Data Science, Human Technopole, Milan, Italy; 5 Department of Cardiothoracic, Vascular Anesthesia and Intensive Care, IRCCS Policlinico San Donato, San Donato Milanese, Milan, Italy; 6 Department of Biomedical Sciences for Health, University of Milan, Milan, Italy

**Keywords:** Physiology, Cardiology, Health occupations, Risk factors

## Abstract

A reduced nocturnal cardiac vagal modulation has been observed in working women with preschoolers. Whether this adaptation also occurs in men remains an open question. The aim of this study was to analyze the cardiac autonomic profile of two groups of healthcare male professionals, one with and one without preschoolers, to be compared to females. Twenty-five working men with preschoolers (M_KID, age 35.41 ± 4.01 years) and 25 without (M_NOKID, 34.48 ± 6.00 years) were compared with 25 working women with preschoolers (W_KID, 37.7 ± 5.6 years) and 25 without (W_NOKID, 35.4 ± 7.2 years). A 24-h Holter electrocardiogram was performed for time and frequency domain analysis of the beat-to-beat variations of RR interval (RR) variability, during daytime (DAY) and nighttime (NIGHT). The power of RR variability in the high frequency band (HF_RR_) was considered as an index of cardiac vagal modulation. RR variability indices were similar in M_KID and M_NOKID during both DAY and NIGHT. In contrast, W_KID showed a reduced nocturnal HF_RR_ compared to W_NOKID. The comparison of working men with and without preschoolers revealed no differences in the cardiac autonomic profile, in contrast with women. This suggests that sex and/or gender may represent a crucial factor in the cardiac neural control in the parental condition.

## Introduction

Nowadays, one of the main challenges of individuals is to find a balance between work duties and those of private life. This could be even harder in presence of young children, above all in particular working settings. Indeed, the presence of preschool children induces physiological changes in working female healthcare professionals, as demonstrated in a recent study^1^, where the cardiac autonomic profile (CAP) of participants was evaluated by means of the heart rate variability analysis. In depth, reduced nocturnal cardiac vagal modulation was observed in female healthcare professionals with preschool children compared to their childless colleagues^1^. This modification of the CAP has been interpreted as physiological, i.e. with a finalistic purpose facilitating a prompt reaction in case of a child’s need.

However, a decreased cardiac vagal modulation has also been associated to an increased risk for overall cardiovascular disease^2–5^ and it has been demonstrated that therapeutic interventions aimed at increasing the cardiac vagal modulation positively affect prognosis^6^. For these reasons, any CAP perturbation should possibly be investigated and considered in terms of primary and secondary prevention.

While it is known that the presence of preschoolers influences the CAP of healthcare female workers, none is known about men. While there is a large number of studies exploring the different impact of family burden on the psycho-social sphere of parents^7–9^, the influence of sex and/or gender on the CAP modification remains an open question. On one hand, women and men have different biological and physiological characteristics that are also expressed at the cardiovascular control level, on the other hand they have different socially constructed norms, behaviors and roles that could impact on the different response of the CAP to external stimuli^7–11^. In this perspective, dealing with preschoolers would represent a strong external stimulus.

Therefore, the first aim of this study was to compare the CAP of a group of working men with preschoolers to a group without, considering both nighttime and daytime, as it has already been investigated in women^1^. The second aim was to compare the CAP of male and female groups in relation to the presence of preschoolers during both nighttime and daytime.

## Methods

### Population

The study population was composed by two groups of male healthcare professionals, one with at least one preschooler (M_KID) and one without (M_NOKID). The sample size of the study was calculated based on a previous study on women with and without preschooler conducted by Dalla Vecchia et al.^1^, assuming a power of 0.90 and a level of significance of 0.05. The derived sample size was 25 subjects for each group. The female population of the cited study^1^ was considered in the present study for comparison: 50 female healthcare professionals, 25 women with at least one preschooler (W_KID, age 37.7 ± 5.6 years) and 25 without (W_NOKID, age 35.4 ± 7.2 years)^1^.

Prior to the enrollment in the study, a screening visit was fixed to verify the inclusion and exclusion criteria. Inclusion criteria were: (1) age between 25 and 45 years; (2) full-time healthcare professional (nurse, physician, physiotherapist or nursing assistant); (3) normal arterial blood pressure (systolic blood pressure ≤ 120–129 mmHg, diastolic blood pressure ≤ 80–84 mmHg)^12^. Exclusion criteria were: (1) any cardiovascular, metabolic, respiratory or intercurrent acute disease; (2) regular intake of medications; (3) moderate to heavy smoking (8 cigarettes/day)^13^; (4) heavy alcohol assumption (more than 250 ml of wine/day or more than 660 ml of beer per day or more than 80 ml of spirits per day)^14^.

### Experimental protocol

The experimental protocol was conducted at IRCCS Istituti Clinici Scientifici Maugeri in Milan. The protocol adhered to the principles of the Declaration of Helsinki and was approved by the ethics committee of Istituti Clinici Scientifici Maugeri IRCCS in Pavia (number of approvals 2131CE, date of approvals 13/06/2017). Each subject signed a written informed consent for the participation to the study protocol.

At enrollment, a detailed clinical evaluation was performed. Right after, a 24-h Holter electrocardiogram (ECG) recording (3 leads, 360° eMotion FAROS, Mega Electronics, Finland) during a regular working day was performed. The recordings were always scheduled after a well slept night, at least 48 h from a bout of heavy physical exercise and a night shift, factors that are known to modify the CAP^15,16^. We also asked the participants to avoid alcoholic and caffeinated beverages in the 24 h preceding and during the ECG recordings and to go to bed by midnight. All the diurnal and nocturnal activities were annotated in a diary.

### Demographic, anamnestic and clinical variables

At the enrollment visit, demographic, anamnestic, and clinical data were collected and participants underwent a complete physical examination, blood pressure measurement and a standard 12-lead ECG. Data included age, sex, sleeping, smoking and alcoholic habits, hours dedicated to regular physical exercise and social activities during the week, and working habits (type of working, night and holiday shifts and hours of work per week). Each subject rated the perceived stress utilizing a visual analogue scale (VAS), an unmarked ruler ranging from 0 (no stress) to 10 (extremely high stress)^17,18^.

### Cardiac autonomic profile characterization

The CAP was assessed by the analysis of the variability of heart period, as derived from the time distance between two consecutive R-wave peaks (RR) derived from 24-h Holter ECG monitoring during a regular working day.

From the 24-h ECG trace derived from a modified lead II, the RR time series were derived. Each RR was calculated as the temporal distance, expressed in ms, between two consecutive R peaks. The algorithm applied for R peak detection was based on a threshold of the first derivative of the ECG signal and allowed to fix R-wave peaks by means of parabolic interpolation. The R-wave peak detections were visually checked to avoid misidentification. In presence of extrasystoles, the RR was corrected by means of cubic spline interpolation. Attention was paid to correct no more than 5% of the total RRs.

For each subject, two periods of 5000 consecutive RRs were chosen for further analyses, one during daytime (DAY, from 1 to 5 pm) and one during nighttime (NIGHT, from 1 to 4 am). An iterated analysis was implemented on the selected periods, i.e. all the parameters described in the following paragraph were calculated on windows of 250 consecutive RRs, with superposition of 200 RRs. The median of their distribution was taken as representative for each considered parameter^19^.

To characterize the CAP of the enrolled population, time and frequency domain indices were calculated over the derived time series. After linear detrending of the RR series, time domain indices were calculated, i.e. mean (μ_RR_) and variance (σ^2^_RR_) of the selected period of RR series during DAY and NIGHT. μ_RR_ and σ^2^_RR_ were expressed in ms and ms^2^, respectively.

The frequency domain parameter was derived by parametric power spectral analysis of the RR series. RR series were modelized as an autoregressive model whose coefficients were estimated via Levinson-Durbin recursion and whose order was optimized by Akaike information criterion. The model order ranged from 8 to 16. The sum of the power spectral components whose central frequency dropped in the high frequency band (HF, 0.15–0.4 Hz)^20^ was taken as an index of the cardiac vagal modulation directed to the sinus node^21,22^ and labelled as HF_RR_. HF_RR_ was expressed in ms^2^.

### Statistical analysis

**Figure 1:**
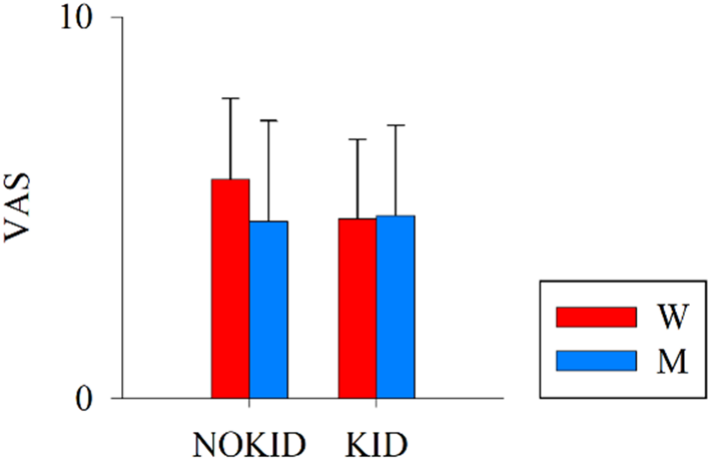
Results of the comparison of the perceived degree of stress, measured by the Visual Analogue Scale, between working men (M, blue bars) and women (W, red bars), with preschoolers (KID) and without (NOKID).

Continuous data were presented as mean ± standard deviation in Table 1 and Fig. 1, otherwise as median (interquartile range). The interquartile range was calculated as the difference between the first and the third quartile. Categorical variables were presented as absolute number (percentage). Normality of the distributions was tested by Kolmogorov–Smirnov test. Demographic, anamnestic and clinical characteristics were compared by two-way analysis of variance or χ^2^ test in case of continuous or categorical variables, respectively.

**Table 1:** Comparison of demographic, anamnestic and clinical features in males and females with and without preschoolers.

	M_NOKID (n = 25)	M_KID (n = 25)	W_NOKID (n = 25)	W_KID (n = 25)
Age, years	34.48 ± 6.00	35.41 ± 4.01	35.4 ± 7.2	37.7 ± 5.6
BMI, kg/m^2^	24.22 ± 2.93	27.86 ± 12.56	22.7 ± 3.7 $#	23.4 ± 3.1$#
Sleep hours per night, hours	6.19 ± 1.16	6.11 ± 0.84	6.6 ± 0.9	6.4 ± 1.3
Light smokers, n (%)	9 (36)	5 (20)	7 (28)	5 (20)
Regular physical exercise, n (%)	15 (60)	10 (40)	12 (48)	8 (32)
Physical exercise, hours/week	4.93 ± 2.47	3.06 ± 2.47	3.0 ± 1.6	2.7 ± 1.9
Regular social activities, n (%)	21 (84)	23 (92)	23 (92)	17 (68)
Social activities, hours/week	5.5 ± 4.44	4.69 ± 3.78	7.4 ± 8.0	3.3 ± 2.3
Working hours per day, hours	7.67 ± 0.93	7.51 ± 1.03	7.9 ± 1.0	7.7 ± 1.4
Night shifts, n (%)	13 (52)	17 (68)	8 (32)	12 (48)
Holiday shifts, n (%)	14 (56)	17 (68)	12 (48)	14 (56)
One child, n (%)		10 (40)		12 (48)
Two children, n (%)		15 (60)		13 (52)
Children age, months		32.16 ± 19.33		33.6 ± 18.1
Working experience, yrs	9.8 ± 5.8	11.2 ± 5.2	10.9 ± 5.7	12.6 ± 4.5
Employment				
Nurse, n (%)	15 (60)	12 (48)	7 (28)	10 (40)
Physician, n (%)	4 (16)	5 (20)	4 (16)	6 (24)
Physiotherapist, n (%)	4 (16)	7 (28)	13 (52)	6 (24)
Nursing assistant, n (%)	2 (8)	1 (4)	1 (4)	3 (12)

M_NOKID, men without children in preschool age; M_KID, men with children in preschool age; W_NOKID, women without children in preschool age; W_KID, women with children in preschool age; BMI, body mass index. Continuous data are presented as mean ± standard deviation, while categorical one as absolute number (percentage). $ indicates *p* < 0.05 versus M_KID; # indicates *p* < 0.05 versus M_NOKID.

As to the primary outcome, two-way repeated measures analysis of variance (ANOVA, Holm-Sidak test for multiple comparisons) was performed to verify the differences between M_KID and M_NOKID within each of the considered experimental condition (i.e. DAY and NIGHT).

The same model was applied to assess differences between experimental conditions (i.e. DAY and NIGHT) in each group (Tables 3, 4) and between gender in each experimental condition (Tables 5, 6). In case of non-normal distribution, the results of the two-way ANOVA were checked performing Kruskal–Wallis one-way ANOVA on ranks (or Friedman test in case of paired samples) separately for each of the two considered ways.

The statistical analyses were carried out using the commercial software Sigmaplot (Systat Software, Inc., Chicago, IL, version 11.0).

## Results

### Results of demographic, anamnestic and clinical variables

A total of 53 male subjects were screened, 3 resulted in a screening failure (2 had hypertension, 1 diabetes), 50 healthy male subjects were finally enrolled in the study and split into M_NOKID and M_KID group (25 subjects each).

M_NOKID included 15 nurses (60%), 4 physicians (16%), 4 physiotherapists (16%), and 2 nursing assistants (8%), while M_KID 12 nurses (48%), 5 physicians (20%), 7 physiotherapists (28%), and 1 nursing assistant (4%).

The results of the demographic, anamnestic and clinical variables of the enrolled male population are shown in Table 1, together with those of the female group investigated in our recent study^1^. All the considered variables were not different between M_NOKID and M_KID. Of notice, the weekly hours dedicated to physical exercise were about two hours higher in M_NOKID than in M_KID, although the difference did not reach any statistical significance. Demographic, clinical and anamnestic characteristics of W_KID and W_NOKID were also similar to each other. No differences were found with respect to male and female characteristics, except for a lower body mass index in women than in men, which is a physiological difference.

As for M_KID, in 40% of cases there was an only child, in the remaining 60% of cases 2 children. The overall mean age of the children was 32.16 ± 19.33 months. In W_KID these data were comparable.

The perceived degree of stress was similar in the two male groups, with a VAS of 4.64 ± 2.64 and 4.80 ± 2.36 in M_NOKID and M_KID, respectively, similarly to women^1^. Figure 1 shows the results of the evaluation of the perceived level of stress of M_NOKID and M_KID compared to W_NOKID and W_KID, respectively. No statistically significant differences were detected.

### Results of cardiac autonomic profile characterization

All the studied subjects reported a well slept night during the Holter ECG recording. The analyses during nighttime were conducted over sleeping periods, according to the individual diary.

Table 2 shows the results of the CAP evaluated in M_KID and M_NOKID groups. μ_RR_, σ^2^_RR_ and HF_RR_ increased from DAY to NIGHT in both M_KID and M_NOKID. μ_RR_ was higher in M_NOKID with respect to M_KID during DAY. No additional differences were observed between M_NOKID and M_KID for any of the considered indices.

**Table 2:** Results of the cardiac autonomic profile comparison in M_NOKID and M_KID during DAY and NIGHT.

	DAY		NIGHT	
	M_NOKID	M_KID	M_NOKID	M_KID
µ_RR_, ms	776.49 (152.86)	691.12 (176.81)#	985.83 (170.15)*	931.10 (203.93)*
σ^2^_RR_, ms^2^	2994.96 (24,769)	1901.91 (2415.53)	4397.23 (5325.48)*	3466.52 (3812.89)*
HF_RR_, ms^2^	297.85 (385.58)	121.62 (102.53)	812.76 (1252.82)*	860.64 (1330.09)*

M_NOKID, men without children in preschool age; M_KID, men with children in preschool age; DAY, daytime; NIGHT, nighttime; RR, RR interval; μ_RR_, mean RR; σ^2^_RR_, variance of RR; HF, high frequency; HF_RR_, power of RR series in the HF band. Results are presented as median. In bracket the interquartile range, calculated as the difference between the first and the third quartile is presented. * indicates *p* < 0.05 DAY versus NIGHT, #indicates *p* < 0.05 M_NOKID versus M_KID.

Table 3 shows the results of the comparison of the CAP between M_NOKID and W_NOKID. μ_RR_, σ^2^_RR_ and HF_RR_ increased from DAY to NIGHT in both groups, independently from sex. No differences were observed between M_NOKID and W_NOKID for any of the considered indices.

**Table 3:** Results of the cardiac autonomic profile comparison between W_NOKID and M_NOKID during DAY and NIGHT.

	DAY		NIGHT	
	W_NOKID	M_NOKID	W_NOKID	M_NOKID
µ_RR_, ms	708.46 (117.19)	776.49 (152.86)	957.04 (221.54)*	985.83 (170.15)*
σ^2^_RR_, ms^2^	2960.54 (2329.65)	2994.96 (24,769)	3295.30 (3983.87)*	4397.23 (5325.48)*
HF_RR_, ms^2^	266.39 (394.96)	297.85 (385.58)	655.68 (1015.02)*	812.76 (1252.82)*

DAY, daytime; NIGHT, nighttime; W_NOKID, women without children in preschool age; M_NOKID, men without children in preschool age; RR, RR interval; μ_RR_, mean RR; σ^2^_RR_, variance of RR; HF, high frequency; HF_RR_, power of RR series in the HF band. Results are presented as median. In bracket the interquartile range, calculated as the difference between the first and the third quartile is presented. * indicates *p* < 0.05 DAY versus NIGHT.

Table 4 shows the results of the comparison between M_KID and W_KID. μ_RR_ increased from DAY to NIGHT in both W_KID and M_KID. σ^2^_RR_ and HF_RR_ increased from DAY to NIGHT in M_KID but not in W_KID. Indeed, HF_RR_ was higher in M_KID compared to W_KID during NIGHT.

**Table 4:** Results of the cardiac autonomic profile comparison between W_KID and M_KID during DAY and NIGHT.

	DAY		NIGHT	
	W_KID	M_KID	W_KID	M_KID
µ_RR_, ms	666.46 (126.33)	691.12 (176.81)	870.52 (221.86)*	931.10 (203.93)*
σ^2^_RR_, ms^2^	2008.57 (1377.48)	1901.91 (2415.53)	1155.73 (1546.36)	3466.52 (3812.89)*
HF_RR_, ms^2^	91.65 (134.97)	121.62 (102.53)	224.68 (483.87)	860.64 (1330.09)*#

DAY, daytime; NIGHT, nighttime; W_KID, women with children in preschool age; M_KID, men with children in preschool age; RR, RR interval; μ_RR_, mean RR; σ^2^_RR_, variance of RR; HF, high frequency; HF_RR_, power of RR series in the HF band. Results are presented as median. In bracket the interquartile range, calculated as the difference between the first and the third quartile is presented. * indicates *p* < 0.05 DAY versus NIGHT; # indicates *p* < 0.05 M_KID versus W_KID.

Table 5 shows the results of the comparison between male and female workers in relation to the presence of preschoolers during DAY. μ_RR_ was lower in M_KID compared to M_NOKID. No differences were detected between women and men for none of the considered index.

**Table 5:** Results of the cardiac autonomic profile comparison between working women and men in relation to the presence of preschoolers during DAY.

	NOKID		KID	
	W	M	W	M
µ_RR_, ms	708.46 (117.19)	776.49 (152.86)	666.46 (126.33)	691.12 (176.81)$
σ^2^_RR_, ms^2^	2960.54 (2329.65)	2994.96 (24,769)	2008.57 (1377.48)	1901.91 (2415.53)
HF_RR_, ms^2^	266.39 (394.96)	297.85 (385.58)	91.65 (134.97)	121.62 (102.53)

DAY, daytime; KID, children in preschool age; NOKID, no children in preschool age; W, women; M, men; RR, RR interval; μ_RR_, mean RR; σ^2^_RR_, variance of RR; HF, high frequency; HF_RR_, power of RR series in the HF band. Results are presented as median. In bracket the interquartile range, calculated as the difference between the first and the third quartile is presented. $ indicates *p* < 0.05 KID versus NOKID.

Table 6 shows the results of the comparison between working women and men in relation to the presence of preschoolers during NIGHT. σ^2^_RR_ and HF_RR_ were lower in W_KID than in W_NOKID and were higher in M_KID compared to W_KID.

**Table 6:** Results of the cardiac autonomic profile comparison between working women and men in relation to the presence of preschoolers during NIGHT.

	NOKID		KID	
	W	M	W	M
µ_RR_, ms	957.04 (221.54)	985.83 (170.15)	870.52 (221.86)	931.10 (203.93)
σ^2^_RR_, ms^2^	3295.30 (3983.87)	4397.23 (5325.48)	1155.73 (1546.36)$	3466.52 (3812.89)£
HF_RR_, ms^2^	655.68 (1015.02)	812.76 (1252.82)	224.68 (483.87)$	860.64 (1330.09)£

DAY, daytime; KID, children in preschool age; NOKID, no children in preschool age; W, women; M, men; RR, RR interval; μ_RR_, mean RR; σ^2^_RR_, variance of RR; HF, high frequency; HF_RR_, power of RR series in the HF band. Results are presented as median. In bracket the interquartile range, calculated as the difference between the first and the third quartile is presented. $ indicates *p* < 0.05 KID versus NOKID; £ indicates *p* < 0.05 M versus W.

The p values related to the analyses shown in Tables 3, 4, 5 and 6 are reported in the supplementary material (Tables S1, S2, S3 and S4, respectively). As for the interaction terms, non-statistically significant results were found except for the HF_RR_ index shown in Table 4 (*p* = 0.026). Notably, in Table 6 the *p* value for the HF_RR_ index was 0.089.

## Discussion

The aim of the present study was to investigate the response of the cardiac autonomic control of male healthcare professionals to the presence of preschool children by comparing the diurnal and nocturnal RR variability indices of a group of men with preschoolers and one without. The results were also compared to those obtained in female healthcare professionals of same age with and without preschoolers.

The main findings of the study can be summarized as follows: (1) the cardiac autonomic profile of men was not influenced by the presence of preschoolers; (2) this result is in contrast with what has been demonstrated in women, in whom the presence of preschoolers induced a decreased nocturnal cardiac vagal modulation^1^; (3) the perceived stress was similar in all the studied groups.

These results originally support the hypothesis that men and women might be differently involved in the management of the preschool offspring. Indeed, contrary to females, cardiac autonomic profile of males with and without preschoolers was not different, either during the daytime or the nighttime. Indeed, σ^2^_RR_ and HF_RR_ were similar between M_NOKID and M_KID both during DAY and NIGHT, suggesting that both groups were characterized by similar vagal cardiac modulation. Different results were observed when the cardiac autonomic profile of working women with preschoolers was compared to that of women without children^1^, a decreased vagal cardiac modulation was observed in the formers.

The comparison between women and men without preschoolers revealed no differences in the CAP, with similar heart rate and vagal cardiac modulation. Despite weak interaction, the comparison between women and men with preschoolers revealed a decreased cardiac vagal modulation during nighttime in women compared to men, as mirrored by a lower HF_RR_ during NIGHT in W_KID compared to M_KID. Thus, the results obtained by the comparison between male and female subjects, representing a secondary aim of the study, need to be confirmed by future research. Indeed, the high number of considered factors could have impacted the statistical power, possibly masking the significance of the interactions between factors, and preventing from definitive conclusions. However, the observed tendencies suggest that the nocturnal period seems to be more interesting than the daily one in evaluating the sex/gender related behavior in presence of preschoolers.

Of notice, these findings were found in presence of similar demographic and clinical variables, including the perceived degree of stress, in all groups. Therefore, neither stress nor other variables seemed to affect the results^23,24^.

Whether merely sex or rather gender plays a role in determining the modification of the cardiac autonomic regulation in presence of preschoolers remains to be established. The observed different behavior in women and men could be ascribable to a different engagement of mothers and fathers in the management of the baby^25^. Indeed, beyond the undisputed biological differences between women and men, and the fact that both motherhood and fatherhood represent a deep and complex experience, the involvement of women in the baby’s life is more all-encompassing than that of men in most socio-cultural contexts, in particular for working women^10,26^. Along with the increase of dual-earner families, fathers are spending more time in the management of the family, however some practical tasks remain more frequently the responsibility of women, or almost exclusively as in the case of breastfeeding^10,11,26^. In addition, the amount and quality of time spent by the parents with the children varies along their growth: in the first years, mothers are often more involved in multitasking activities, often suffering the pressure of rigid timetables, work and family duties^10^. Fathers may tend to engage in a more physical play, while mothers often demonstrate greater responsiveness to infant cues^10,26^. These attitude and psychological differences might contribute in determining the different CAP response, where the reduced maternal cardiac vagal modulation at night may regulate behavioural, cognitive and emotional responses by inhibiting the central autonomic network^27^. Given the different engagement in the children care, the different adaptation of the CAP found in mothers and fathers ultimately reinforces the concept that the decreased vagal modulation in women might be the autonomic response to a stressor that has some finalistic purpose. It is difficult to ascertain whether this physiological response carries any advantage, practical for the baby or physiological for the mother, or it is simply an ancestral relic that could indeed have a prognostically negative effect on women.

Interestingly, comparing M_NOKID to M_KID, a longer μ_RR_ during DAY was observed in the former group. An analogous but not significant tendency towards a longer μ_RR_ was present also during NIGHT. It can be assumed that the reason could be a higher level of physical training in M_NOKID^28^, in fact the percentage of men regularly exercising in the two groups was 20% higher in M_NOKID with an average of 2 h longer of training per week. It should be noted that this single difference between the two groups may represent a minor limitation of the study. Remarkably, this possible bias did not lead to significant variation between vagal modulation as estimated via the amplitude of respiratory sinus arrhythmia, thus indicating the minor impact of this limitation.

Unfortunately, accurate and detailed data on sleep and stress assessment are not available, which is certainly a further limitation. However, the evidence emerging from the present study suggests that there is an important role of sex or gender, or both, in cardiac autonomic control responses. These influences are certainly to be considered and deepened in future studies exploring the physiological or pathophysiological mechanisms and their possible prognostic significance in primary and secondary prevention. Future studies should also deepen the relationship between the modification induced by fatherhood and motherhood on the CAP of different professional categories.

## Conclusion

In the present study, we compared a group of working men with preschool children to a group without. The demographic and clinical characteristics were similar in the two groups, there were no differences in terms of perceived stress level and no difference in the cardiac autonomic profile. This latter finding is in contrast with what has been shown in women; in fact, working mothers are characterized by a different CAP compared to women without young children. This evidence suggests that sex and/or gender may represent a crucial factor in the cardiac neural control in parenthood.

## Supplementary Information

Supplementary Tables.

## Data Availability

Data are available upon reasonable request to the corresponding author.

## References

[1] Dalla Vecchia LA (2021). How the first years of motherhood impact the cardiac autonomic profile of female healthcare professionals: A study by heart rate variability analysis. Sci. Rep..

[2] La-Rovere MT, Bigger JT, Marcus FI, Mortara A, Schwartz PJ (1998). Baroreflex sensitivity and heart-rate variability in prediction of total cardiac mortality after myocardial infarction. ATRAMI (Autonomic Tone and Reflexes After Myocardial Infarction) investigators. Lancet.

[3] Pinna GD (2017). Different estimation methods of spontaneous baroreflex sensitivity have different predictive value in heart failure patients. J. Hypertens..

[4] Saxena T, Ali AO, Saxena M (2018). Pathophysiology of essential hypertension: An update. Expert Rev. Cardiovasc. Ther..

[5] Sessa F (2018). Heart rate variability as predictive factor for sudden cardiac death. Aging (Albany NY).

[6] Dalla Vecchia L (2013). Favorable effects of carotid endarterectomy on baroreflex sensitivity and cardiovascular neural modulation: A 4-month follow-up. Am. J. Physiol. Regul. Integr. Comp. Physiol..

[7] Koenig J, Thayer JF (2016). Sex differences in healthy human heart rate variability: A meta-analysis. Neurosci. Biobehav. Rev..

[8] Shaffer F, Ginsberg JP (2017). An overview of heart rate variability metrics and norms. Front. Public Health..

[9] Backholer K (2017). Sex differences in the relationship between socioeconomic status and cardiovascular disease: A systematic review and meta-analysis. J. Epidemiol. Community Health.

[10] Craig L (2006). Does father care mean fathers share? A comparison of how mothers and fathers in intact families spend time with children. Gend. Soc..

[11] Schoppe-Sullivan SJ, Kotila L, Jia R, Lang SN, Bower DJ (2013). Comparisons of levels and predictors of mothers' and fathers' engagement with their preschool aged children. Early Child. Dev. Care.

[12] Williams B (2018). Practice guidelines for the management of arterial hypertension of the European Society of Cardiology and the European Society of Hypertension. Blood Press..

[13] Baumert J (2010). Determinants of heavy cigarette smoking: are there differences in men and women? Results from the population-based MONICA/KORA Augsburg surveys. Nicotine Tob. Res..

[14] Stockwell T (2016). Do "moderate" drinkers have reduced mortality risk? A systematic review and meta-analysis of alcohol consumption and all-cause mortality. J. Stud. Alcohol. Drugs.

[15] Dalla Vecchia L, Traversi E, Porta A, Lucini D, Pagani M (2014). On site assessment of cardiac function and neural regulation in amateur half marathon runners. Open Heart.

[16] Shiffer D (2018). Effects of clockwise and counterclockwise job shift work rotation on sleep and work-life balance on hospital nurses. Int. J. Environ. Res. Public. Health..

[17] Lesage FX, Berjot S, Deschamps F (2012). Clinical stress assessment using a visual analogue scale. Occup. Med. (Lond.).

[18] Mitchell AM, Crane PA, Kim Y (2008). Perceived stress in survivors of suicide: Psychometric properties of the Perceived Stress Scale. Res. Nurs. Health.

[19] Porta A (2007). An integrated approach based on uniform quantization for the evaluation of complexity of short-term heart period variability: Application to 24 h Holter recordings in healthy and heart failure humans. Chaos.

[20] Camm AJ (1996). Heart rate variability. Standards of measurement, physiological interpretation, and clinical use. Task Force of the European Society of Cardiology and the North American Society of Pacing and Electrophysiology. Eur. Heart J..

[21] Pagani M (1986). Power spectral analysis of heart rate and arterial pressure variabilities as a marker of sympatho-vagal interaction in man and conscious dog. Circ. Res..

[22] Akselrod S (1981). Power spectrum analysis of heart rate fluctuation: A quantitative probe of beat-to-beat cardiovascular control. Science.

[23] Carnevali L, Sgoifo A (2014). Vagal modulation of resting heart rate in rats: The role of stress, psychosocial factors, and physical exercise. Front. Physiol..

[24] Somers VK, Dyken ME, Mark AL, Abboud FM (1993). Sympathetic-nerve activity during sleep in normal subjects. N. Engl. J. Med..

[25] Rajhans P, Goin-Kochel RP, Strathearn L, Kim S (2019). It takes two! Exploring sex differences in parenting neurobiology and behaviour. J. Neuroendocrinol..

[26] Lang SN (2014). Relations between fathers' and mothers' infant engagement patterns in dual-earner families and toddler competence. J. Fam. Issues.

[27] Balzarotti S, Biassoni F, Colombo B, Ciceri MR (2017). Cardiac vagal control as a marker of emotion regulation in healthy adults: A review. Biol. Psychol..

[28] Dalla Vecchia LA (2019). Can strenuous exercise harm the heart? Insights from a study of cardiovascular neural regulation in amateur triathletes. PLoS ONE.

