# Supplementary material for: Autonomic cardiac profile in male and female healthcare professionals with and without preschoolers: differences evidenced by heart rate variability analysis
Source: Sci Rep. 2022 Aug 25;12:14499. doi: 10.1038/s41598-022-18744-1 (PMC9411539; doi:10.1038/s41598-022-18744-1)
Supplement: Supplementary file 1 — Supplementary Tables. [file 41598_2022_18744_MOESM1_ESM.docx]

**Table S1. Results of the cardiac autonomic profile comparison between W_NOKID and M_NOKID during DAY and NIGHT**

|  | DAY | | NIGHT | |  | |  | |  |
| --- | --- | --- | --- | --- | --- | --- | --- | --- | --- |
|  | W_NOKID | M_NOKID | W_NOKID | M_NOKID | | p M vs W | | p DAY vs NIGHT | p interactions |
| µ_RR_, ms | 708.46 (117.19) | 776.49 (152.86) | 957.04 (221.54)* | 985.83 (170.15)* | | 0.181 | | <0.001 | 0.941 |
| σ^2^_RR_, ms^2^ | 2960.54 (2329.65) | 2994.96 (24769) | 3295.30 (3983.87)* | 4397.23 (5325.48)* | | 0.818 | | 0.006 | 0.805 |
| HF_RR_, ms^2^ | 266.39 (394.96) | 297.85 (385.58) | 655.68 (1015.02)* | 812.76 (1252.82)* | | 0.626 | | <0.001 | 0.536 |

DAY, daytime; NIGHT, nighttime; W_NOKID, women without children in preschool age; M_NOKID, men without children in preschool age; RR, RR interval; μ_RR_, mean RR; σ^2^_RR_, variance of RR; HF, high frequency; HF_RR_, power of RR series in the HF band. Results are presented as median. In bracket the interquartile range, calculated as the difference between the first and the third quartile is presented. * indicates p<0.05 DAY vs NIGHT.

**Table S2. Results of the cardiac autonomic profile comparison between W_KID and M_KID during DAY and NIGHT**

|  | DAY | | NIGHT | |  |  |  |
| --- | --- | --- | --- | --- | --- | --- | --- |
|  | W_KID | M_KID | W_KID | M_KID | p M vs W | p DAY vs NIGHT | p interactions |
| µ_RR_, ms | 666.46 (126.33) | 691.12 (176.81) | 870.52 (221.86)* | 931.10 (203.93)* | 0.584 | <0.001 | 0.255 |
| σ^2^_RR_, ms^2^ | 2008.57 (1377.48) | 1901.91 (2415.53) | 1155.73 (1546.36) | 3466.52 (3812.89)* | 0.098 | 0.055 | 0.100 |
| HF_RR_, ms^2^ | 91.65 (134.97) | 121.62 (102.53) | 224.68 (483.87) | 860.64 (1330.09)*# | 0.074 | <0.001 | 0.026 |

DAY, daytime; NIGHT, nighttime; W_KID, women with children in preschool age; M_KID, men with children in preschool age; RR, RR interval; μ_RR_, mean RR; σ^2^_RR_, variance of RR; HF, high frequency; HF_RR_, power of RR series in the HF band. Results are presented as median. In bracket the interquartile range, calculated as the difference between the first and the third quartile is presented. * indicates p<0.05 DAY vs NIGHT; # indicates p<0.05 M_KID vs W_KID.

**Table S3. Results of the cardiac autonomic profile comparison between working women and men in relation to the presence of pre-schoolers during DAY**

|  | NOKID | | KID | |  |  |  |
| --- | --- | --- | --- | --- | --- | --- | --- |
|  | W | M | W | M | p M vs W | p KID vs NOKID | p interactions |
| µ_RR_, ms | 708.46 (117.19) | 776.49 (152.86) | 666.46 (126.33) | 691.12 (176.81)$ | 0.317 | 0.007 | 0.204 |
| σ^2^_RR_, ms^2^ | 2960.54 (2329.65) | 2994.96 (24769) | 2008.57 (1377.48)$ | 1901.91 (2415.53) | 0.847 | 0.004 | 0.737 |
| HF_RR_, ms^2^ | 266.39 (394.96) | 297.85 (385.58) | 91.65 (134.97) | 121.62 (102.53) | 0.647 | 0.094 | 0.631 |

DAY, daytime; KID, children in preschool age; NOKID, no children in preschool age; W, women; M, men; RR, RR interval; μ_RR_, mean RR; σ^2^_RR_, variance of RR; HF, high frequency; HF_RR_, power of RR series in the HF band. Results are presented as median. In bracket the interquartile range, calculated as the difference between the first and the third quartile is presented. $ indicates p<0.05 KID vs NOKID.

**Table S4. Results of the cardiac autonomic profile comparison between working women and men in relation to the presence of pre-schoolers during NIGHT**

|  | NOKID | | KID | |  |  |  |
| --- | --- | --- | --- | --- | --- | --- | --- |
|  | W | M | W | M | p M vs W | p KID vs NOKID | p interactions |
| µ_RR_, ms | 957.04 (221.54) | 985.83 (170.15) | 870.52 (221.86) | 931.10 (203.93) | 0.204 | 0.055 | 0.930 |
| σ^2^_RR_, ms^2^ | 3295.30 (3983.87) | 4397.23 (5325.48) | 1155.73 (1546.36)$ | 3466.52 (3812.89)£ | 0.342 | 0.034 | 0.177 |
| HF_RR_, ms^2^ | 655.68 (1015.02) | 812.76 (1252.82) | 224.68 (483.87)$ | 860.64 (1330.09)£ | 0.414 | 0.139 | 0.089 |

DAY, daytime; KID, children in preschool age; NOKID, no children in preschool age; W, women; M, men; RR, RR interval; μ_RR_, mean RR; σ^2^_RR_, variance of RR; HF, high frequency; HF_RR_, power of RR series in the HF band. Results are presented as median. In bracket the interquartile range, calculated as the difference between the first and the third quartile is presented. $ indicates p<0.05 KID vs NOKID; £ indicates p<0.05 M vs W.
